# Sleep Quality and Factors Influencing Self-Reported Sleep Duration and Quality in the General Internal Medicine Inpatient Population

**DOI:** 10.1371/journal.pone.0156735

**Published:** 2016-06-09

**Authors:** Selina Dobing, Natalia Frolova, Finlay McAlister, Jennifer Ringrose

**Affiliations:** 1 Department of Medicine, University of Alberta, Edmonton, Canada; 2 Division of General Internal Medicine, University of Alberta, Edmonton, Canada; University of Colorado Denver, UNITED STATES

## Abstract

**Background:**

Sleep quality in hospitalized Canadian General Internal Medicine patients is not well characterized. Our goals were to characterize hospital sleep quality in this population and identify potentially modifiable barriers to good sleep.

**Methods:**

GIM inpatients at a quaternary centre in Edmonton, Canada completed a survey, including the Verran-Snyder Halpern (VSH) questionnaire, to characterize the previous night’s sleep within 48 hours prior to discharge. A chart review was also completed to assess comorbidities, discharge diagnoses, and pharmaceutical sleep aid use.

**Results:**

Patients reported significantly worse nighttime sleep duration in hospital compared with home (mean 5.5 versus 7.0 hours per night, p < 0.0001). Sleep quality was poor, as measured by the VSH disturbance (mean 371), effectiveness (190), and supplementation (115) subscales. Variables independently associated with poorer sleep duration in multivariable regression include prior diagnosis of sleep disorder and multi-patient occupancy rooms. Age, sex, admitting diagnosis, length of stay, frequency of vital checks, and use of sleep pharmaceuticals during the index hospitalization were not associated with sleep duration. The most frequently reported reasons for poor sleep included noise (59%), nursing interruptions (30%), uncomfortable beds (18%), bright lights (16%), unfamiliar surroundings (14%), and pain (9%).

**Conclusions:**

Sleep quality for GIM inpatients is significantly worse in hospital than at home. There is a need to test non-pharmacologic interventions to address the most frequently identified factors causing poor sleep hygiene for GIM inpatients.

## Introduction

Insomnia, the subjective disruption of the quantity or quality of sleep, affects 4–38% of North Americans and 6–70% people worldwide [1]. Insomnia is associated with adverse cognitive outcomes (confusion, depression, anxiety, decreased memory, etc.), falls/accidents, and decreased quality of life [2]. Hospitalization can further disrupt sleep due to active medical problems, medications, loss of the normal sleep-wake cycle, decreased daytime activity, and noise [3,4,5]. Due to their medical complexity, general internal medicine (GIM) patients are high risk for both insomnia and complications of insomnia, such as delirium and falls [2,6].

There is a growing body of research on sleep quality and sleep duration in hospitalized patients. Insomnia is prevalent in the medical inpatient population [4]. We are aware of only one Canadian study where family medicine and general medicine patients were grouped together [7]. Other studies have excluded patients transferred from an intensive care unit, those unable to ambulate, those with previously documented sleep disorders or those that are being discharged to a location other than independent community living [8,9].

Assessment of sleep duration and quality has been done using various methods [10]. A recent literature review identified three subjective sleep measurement scales: the Richards-Campbell Sleep Questionnaire, the St. Mary’s Hospital Sleep Questionnaire and the Verran Snyder-Halpern (VSH) Sleep Scale [10]. For this research, the VSH was selected as it has been validated in hospital inpatients in the non-intensive care setting. While this scale is longer than other scales designed to measure sleep quality, it has been validated in our patient population and captures sleep disturbance and total sleep time [10].

Our study is the first to examine the quality and subjective duration of sleep in this Canadian general internal medicine inpatient population and identify potentially modifiable factors that negatively impact their sleep quality. Unlike most sleep assessment studies, we elected to survey patients close to the time of discharge to minimize the influence of acute medical symptoms.

## Methods

### Study Design

This one-month, cross sectional survey was conducted at an 880-bed quaternary centre in Edmonton, Alberta, Canada in March 2015. Approval was obtained from the University of Alberta Research Ethics Board for this research and consent procedure. Written informed consent was obtained from participants prior to participation. As limited data exists on sleep quality in our patient population, we were unable to estimate precision around the variables we planned to collect. We targeted a convenience sample of 100 consecutive eligible participants.

All patients admitted to one of four general internal medicine wards were eligible. Participants had to be 18 years or older, able to communicate in English, and willing/able to consent and complete the questionnaire. We excluded those with a life expectancy < 3 months, with significant cognitive impairment (score of less than 5 on the Short Portable Mental Status Questionnaire) [11], or who had previously enrolled in the study. If the patients did not have significant cognitive impairment, the discharge location (long term care, assisted living, independent living, another institution) was not an exclusion criterion. Patients with pre-existing sleep disorders (sleep apnea, insomnia) were also included.

### Data Collection

Patients were approached within 48 hours of discharge. This timing was chosen to minimize the effect of the acute illness on sleep. Eligible participants completed an English-language questionnaire on perceived sleep quality and factors affecting sleep quality (S1 Questionnaire). Participants also completed the Verran and Snyder-Halpern Sleep Scale (VSH), a validated in-hospital tool that measures the previous night’s sleep characteristics [12]. This is a 15 item visual analogue scale, scored from 0–100 mm, partitioned into three subscales (sleep disturbance, effectiveness and supplementation). A high score indicates worse sleep for the sleep disturbance and supplementation subscales, and better sleep for the sleep effectiveness subscale. A chart review was performed for participant comorbidities, primary reason for hospitalization and sleep pharmaceutical use at home and in hospital. Specific to sleep, our chart review specifically recorded a documented history of: sleep disordered breathing (OSA, OHS, CSA), insomnia, sleep movement disorders, other (text permitted), or chronic sleep aid use as an outpatient.

### Statistical Analysis

All data were analyzed using SAS version 9.4 (Cary, NC) and statistical significance was defined as p<0.05. Multivariate models included age, sex, inpatient sleep aid use, and variables with univariate p-value ≤ 0.15. Patients with missing response variables were excluded from analysis. As there were no significant differences in sleep quality between the 4 GIM wards, all patients were combined for analyses.

## Results

**Fig 1:**
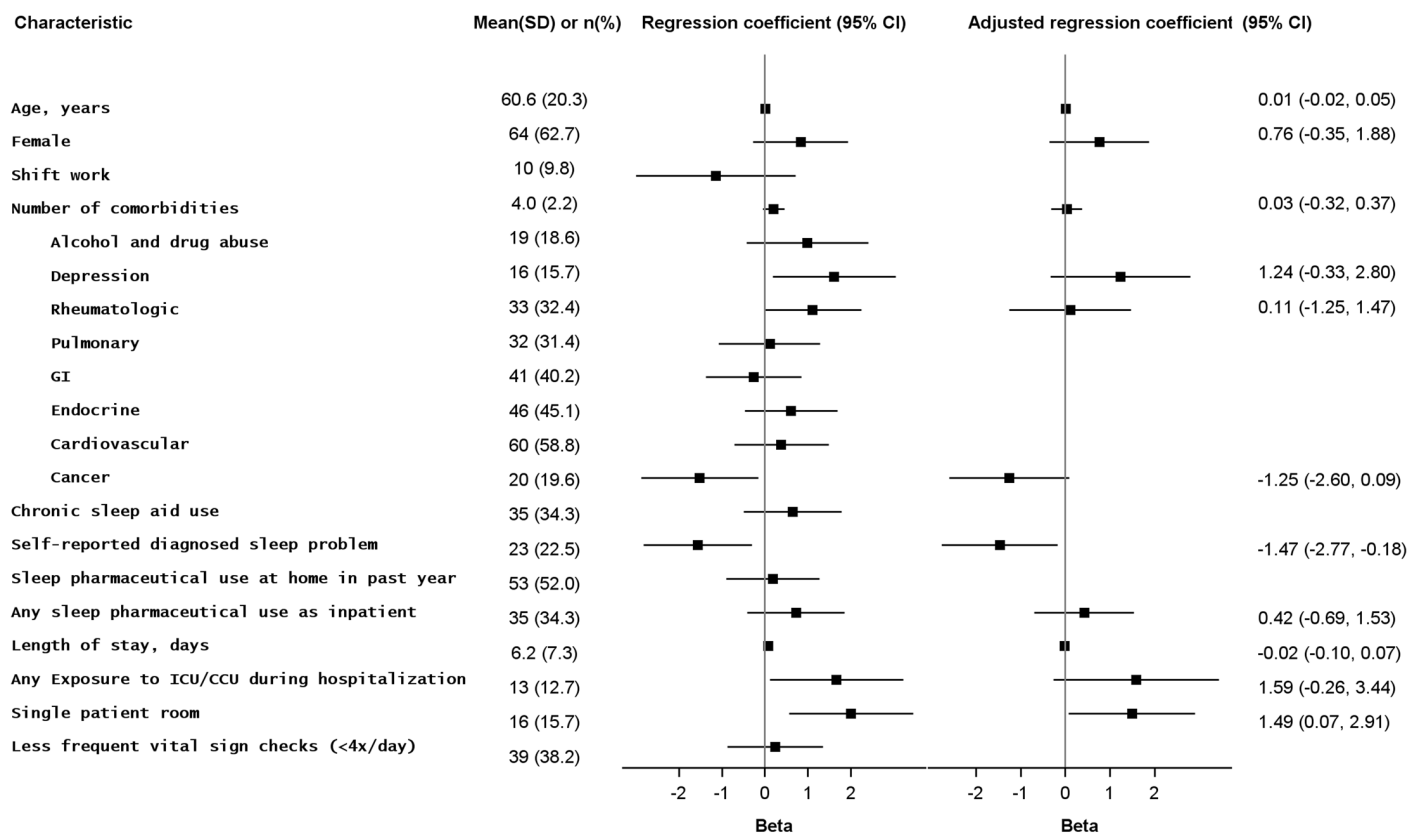
Patient characteristics and associations with factors that may affect sleep. SD = standard deviation; CI = confidence interval. Patient characteristic values are displayed as n (%) for binary variables and mean (SD) for continuous variables.Associations are represented by linear regression coefficients (Beta), displayed for both crude and adjusted models with corresponding 95% confidence intervals. Coefficients greater than zero represent a positive association (i.e., variable is positively correlated with sleep duration in the previous night), while coefficients less than zero represent inverse association (i.e., variable is negatively correlated with sleep duration in the previous night).

During the study period, 154 patients were screened for study eligibility. Of these, 16 failed cognitive impairment screening, 14 were unable to communicate in English, 9 declined participation, 3 were excluded for limited life expectancy, and 10 were eligible but not discharged within 48 hours of completing the survey and were removed from the study. Of the 102 eligible patients, 97 completed the qualitative survey and 93 appropriately completed the VSH scale. Patient characteristics are found in Fig 1. The most common discharge diagnoses were: infection (36.3%), other (24.5%), venous thromboembolism (11.8%), delirium, failure to thrive, or fall (9.8%, diabetes mellitus (6.9%), drug overdose or withdrawal (5.9%), or heart failure (4.9%)

### Sleep Duration and Quality

Overall, patients reported significantly worse sleep in hospital than at home (5.5 [95%CI 5.0–6.0] vs. 7.0 [6.6–7.4], p<0.0001) and more daytime naps (1.6 [95%CI 1.2–1.9] vs. 1.0 [0.8–1.3], p 0.003) in hospital. Quality of sleep in the night prior to survey was poor, as measured by the VSH sleep disturbance, effectiveness, and supplementation subscales (means 371, 190, and 115, respectively) (Table 1). Self-reported duration of the previous night’s sleep was correlated with both VSH disturbance (r = -0.51, p < 0.0001) and effectiveness (r = 0.41, p < 0.0001) subscales.

**Table 1:** Verran Snyder Halpern Sleep Scale scores for inpatients surveyed within 48 hours of discharge (n = 93).

Sleep Characteristic Subscale *	Mean (95% CI)	Median (IQR)	Range
Sleep Disturbance:	371 (335, 406)	395 (223, 509)	20–682
Midsleep awakening	49 (44, 55)	49 (24, 71)	0–97
Wake after sleep onset	64 (57, 70)	76 (40, 89)	1–97
Movement during sleep	47 (40, 54)	46 (14, 79)	0–97
Soundness of sleep	61 (53, 68)	68 (23, 95)	3–100
Quality of disturbance	56 (48, 63)	69 (14, 89)	0–97
Sleep latency †	48 (41, 55)	49 (14, 79)	0–97
Quality of latency	46 (39, 54)	47 (8, 86)	0–97
Sleep Effectiveness:	190 (168, 211)	191 (102, 266)	6–383
Total sleep period	50 (44, 55)	51 (26, 69)	0–97
Rest upon awakening	56 (49, 64)	58 (22, 93)	3–100
Subjective quality of sleep	43 (36, 50)	42 (7, 76)	0–97
Sleep sufficiency evaluation ‡	41 (34, 48)	26 (13, 72)	3–100
Sleep Supplementation:	115 (97, 133)	108 (41, 174)	0–336
Daytime sleep	20 (16, 25)	12 (4, 31)	0–97
Morning sleep	35 (28, 42)	18 (3, 73)	0–97
Evening sleep	31 (24, 39)	10 (2, 73)	0–97
Wake after final arousal §	29 (22, 36)	10 (3, 58)	0–97

*VSH subscales are intended to represent characteristics of sleep quality found in classic sleep taxonomy literature^10^.

†Sleep latency is the amount of time between settling down to sleep and actually falling asleep.

‡Sleep sufficiency evaluation represents a person’s perception of having received sufficient quantity of sleep.

§Wake after final arousal is a person’s ability to remain awake after morning awakening.

### Factors Affecting Sleep Quality

The only variables associated with previous night’s sleep duration on multivariate analysis were prior self-reported diagnosis of sleep disorder (shorter sleep) and single patient room (longer sleep) (Fig 1). Prior diagnosis of self-reported sleep disorder was also independently associated with worse sleep on the VSH sleep disturbance and effectiveness subscales (Fig 1). Sleep pharmaceutical use was not associated with self-reported sleep duration, or the VSH sleep disturbance or effectiveness subscales (Fig 1), and neither were any of the other variables in Fig 1.

### Qualitative Results

When asked to describe factors affecting nighttime sleep quality, the most frequently identified factor was noise (59.2%), with six patients specifically citing noise from staff (8.5%), and twelve citing noise from other patients (16.9%). Other commonly identified factors included nursing care interruptions for vital signs or medication administration (29.6%), uncomfortable beds (18.3%), inappropriately bright lighting (15.5%), unfamiliar surroundings (14.1%), pain (8.5%), anxiety (5.6%), nocturia (2.8%), too much daytime sleep (2.8%), intravenous lines (1.4%), coughing (1.4%), and odours (1.4%).

## Discussion

We have confirmed, in our Canadian, General Internal Medicine inpatient population, that sleep quality and duration is poor. GIM patients are at high risk for insomnia even pre-hospitalization, with 22.5% self-reporting a diagnosed sleep problem. Prior diagnosis of a sleep disorder or sleeping in a multi-patient room were the strongest predictors of poor sleep in hospital.

Use of sleep pharmaceuticals, which have been associated with adverse cognitive outcomes and falls [6], did not correlate with improved sleep quality, however our study design limits the inferences that can be drawn from this due to the very real possibility of confounding by indication.

Environmental factors (e.g. noise, lighting, nursing care interruptions) were more frequently cited causes of poor sleep than medical acuity.

Our survey uptake was over 90%, thus reducing the risk of non-response bias. Our results for reported sleep duration and VSH scores are consistent with previous reports in general medicine patients [7,13, 14]. We also performed a comprehensive survey of factors that may be associated with nighttime sleep quality, using three different quality measurements (Table 1). Our single question, self-reported quantification of the previous night’s sleep, performed similarly to the validated, but more cumbersome, VSH and effectiveness subscales, and thus could serve as a simple screening tool for inpatient sleep duration.

Limitations to this study include reliance on patient recall for their usual sleep duration prior to hospitalization and in hospital. These results may not accurately reflect the effect of acute medical illnesses on sleep quality earlier in the hospital course as we collected data on sleep quality just prior to discharge. Thus, we cannot comment on total sleep deficit accumulated by GIM inpatients.

## Conclusions

GIM inpatients experience significantly worse sleep in hospital than at home. Our results suggest that non-pharmacologic interventions, such as noise reduction policies, lighting protocols, and increased single-patient rooms, should be the focus of future quality improvement processes aimed at improving GIM inpatient sleep quality.

## Supporting Information

S1 Questionnaire (DOCX)
