# Supplementary material for: Sleep Quality and Factors Influencing Self-Reported Sleep Duration and Quality in the General Internal Medicine Inpatient Population
Source: PLoS One. 2016 Jun 9;11(6):e0156735. doi: 10.1371/journal.pone.0156735 (PMC4900612; doi:10.1371/journal.pone.0156735)
Supplement: S1 Questionnaire — (DOCX) [file pone.0156735.s001.docx]

**PROJECT:** Assessing Sleep Quality and Sleep Aid Medication Use in the General Internal Medicine Inpatient Population

Study identifier ____ ___ ____ ____ ____ ____ _____________
 ^Unit # Patient # Patient initials^

Please answer each of the following items as they apply to you. Answer as honestly as you can. Thank you for participating in this study.

**A: General Questions**

1. Before you were admitted to hospital, did you have any regular difficulty with sleeping?

□ Yes □ No

2. Have you ever been diagnosed with a sleep problem?

□ Yes □ No

3a. Have you taken any medications to help with sleep in the past year?

□ Yes □ No → if NO, go straight to #4.

3b. If you answered “YES” for 3a, how many nights on average will you take a medication sleep aid at home?

3c. If you answered “YES” for 3a, how many nights on average did you take a medication sleep aid in hospital?

4. Have you done any shift-based work in the past year?

□ Yes □ No

5a. How does the quality of your sleep in hospital compare with out of hospital (e.g. home)?

□ Better □ Worse □ Same → if SAME, go straight to #6

5b. If you answered “ BETTER” or “WORSE” for #5a, why do you think your sleep in hospital is of better or worse quality?

Please turn to the **next page**.

**PROJECT:** Assessing Sleep Quality and Sleep Aid Medication Use in the General Internal Medicine Inpatient Population

Study identifier ____ ___ ____ ____ ____ ____ _____________
 ^Unit # Patient # Patient initials^

6a. How many hours on average do you sleep at home during nighttime hours?

6b. How many hours on average do you sleep in hospital during nighttime hours?

7a. How many hours on average do you sleep at home during daytime hours (i.e. napping)?

7b. How many hours on average do you sleep in hospital during daytime hours (i.e. napping)?

8. What time did you first fall asleep last night?

9. What time did you wake up this morning?

10. Was your sleep continuous?

Please turn to the **next page**.

**PROJECT:** Assessing Sleep Quality and Sleep Aid Medication Use in the General Internal Medicine Inpatient Population

Study identifier ____ ___ ____ ____ ____ ____ _____________
 ^Unit # Patient # Patient initials^

For the next set of questions, answer by placing an X on the answer line. Place your X anywhere along the line that you feel **best** describes your **last night’s sleep**. The following is an example of the questions you are about to answer:

**EXAMPLE A:**

During my sleep last night, I:

Did not awaken –––––––––––––––––––––––––––––––––––––––––––––––X Was awake 10 hours

If you were awake for 10+ hours, you would put your X at the end of the line, next to the words “Was awake 10 hours.”

**EXAMPLE B:**

During my sleep last night, I:

Did not awaken –––––––––––––––––––––––X–––––––––––––––––––––––– Was awake 10 hours

If you were awake for 5 hours, you would put your X in the middle of the line.

**EXAMPLE C:**

During my sleep last night, I:

Did not awaken X––––––––––––––––––––––––––––––––––––––––––––––– Was awake 10 hours

If you were asleep for the entire night, you would put your X at the start of the line.

Please turn to the **next page**.

**PROJECT:** Assessing Sleep Quality and Sleep Aid Medication Use in the General Internal Medicine Inpatient Population

Study identifier ____ ___ ____ ____ ____ ____ _____________
 ^Unit # Patient # Patient initials^

**Place your X anywhere on the answer line where it best represents your sleep last night:**

Did not awaken ––––––––––––––––––––––––––––––––––––––––––––––– Was awake 10 hours

Had no sleep ––––––––––––––––––––––––––––––––––––––––––––––– Had 10 hours sleep

No sleep during the ––––––––––––––––––––––––––––––––––––––––––––––– Slept 10 hours during

day yesterday the day

Did not sleep ––––––––––––––––––––––––––––––––––––––––––––––– Slept off and on

yesterday morning yesterday morning

Did not sleep ––––––––––––––––––––––––––––––––––––––––––––––– Slept off and on

yesterday evening yesterday evening

Fell asleep ––––––––––––––––––––––––––––––––––––––––––––––– Did not fall asleep

immediately

Slept lightly ––––––––––––––––––––––––––––––––––––––––––––––– Slept deeply

Had no trouble with ––––––––––––––––––––––––––––––––––––––––––––––– Had a lot of trouble

disrupted sleep with disrupted sleep

Didn’t wake at all ––––––––––––––––––––––––––––––––––––––––––––––– Was awake off and on

all night

Had no trouble ––––––––––––––––––––––––––––––––––––––––––––––– Had a lot of trouble

falling asleep falling asleep

Didn’t move ––––––––––––––––––––––––––––––––––––––––––––––– Tossed all night

Please turn to the **next page**.

**PROJECT:** Assessing Sleep Quality and Sleep Aid Medication Use in the General Internal Medicine Inpatient Population

Study identifier ____ ___ ____ ____ ____ ____ _____________
 ^Unit # Patient # Patient initials^

**Place your X anywhere on the answer line where it best represents your sleep last night:**

Awoke refreshed ––––––––––––––––––––––––––––––––––––––––––––––– Awoke exhausted

After morning ––––––––––––––––––––––––––––––––––––––––––––––– After morning

awakening, stayed awake awakening, dozed off

Had a bad night’s ––––––––––––––––––––––––––––––––––––––––––––––– Had a good night’s

sleep sleep

Had enough sleep ––––––––––––––––––––––––––––––––––––––––––––––– Did not have enough

sleep

END OF QUESTIONNAIRE – THANKS!
